# Experts' Views on Factors Influencing Resource Allocation for Infectious Disease Emergencies Based on Humanitarian Principles: A Qualitative Study

**DOI:** 10.1016/j.focus.2024.100286

**Published:** 2024-10-05

**Authors:** Mohammad Reza Fallah Ghanbari, Katayoun Jahangiri, Mehdi Safari, Zohreh Ghomian, Mohammad Ali Nekooie

**Affiliations:** 1 Candidate of Health in Disasters and Emergencies, School of Public Health and Safety, Shahid Beheshti University of Medical Sciences, Tehran, Iran; 2 Faculty of Civil Engineering, Alborz University, Karaj, Iran

**Keywords:** Humanitarian principles, resource allocation, health, infectious diseases, emergencies

## Abstract

**Introduction:**

Infectious diseases can result in global emergencies and a shortage of resources, leading to ethical and humanitarian challenges. This study aimed to identify the factors that affect the allocation of health resources based on humanitarian principles during infectious disease emergencies.

**Methods:**

This study was conducted using a qualitative approach known as content analysis, and 23 specialists and experts with practical experience and theoretical knowledge in this area were carefully selected to participate in the study. The selection process continued until the principle of data saturation was attained. Data collection was done through in-depth, semi-structured interviews that were conducted from October to December 2023.

**Results:**

Factors affecting resource allocation were categorized into 9 main themes, 25 categories, and 50 subcategories. The extracted themes are: (1) Rules and Regulations; (2) Quality in Allocation; (3) Human Aspects; (4) Epidemic/Pandemic Characteristics; (5) Governance and Policymaking; (6) Emergency Management; (7) Resource Management; (8) Solidarity; (9) Trustworthiness.

**Conclusions:**

During emergencies caused by infectious diseases, resource allocation requires attention to various aspects such as laws, protocols, procedures, organizational structures, and coordination mechanisms. Ethical principles and respect for human aspects are also important.

## HIGHLIGHTS

• Resource shortages during infectious disease emergencies can lead to humanitarian challenges.
• Governments are a major component of implementing humanitarian principles.
• Respecting human aspects and ethical issues has an impact on humanitarian principles.
• Trust and transparency enhance impartiality, neutrality, and independence.
• Solidarity is becoming an increasingly important principle in the humanitarian context.

## Graphical abstract

**Figure:**
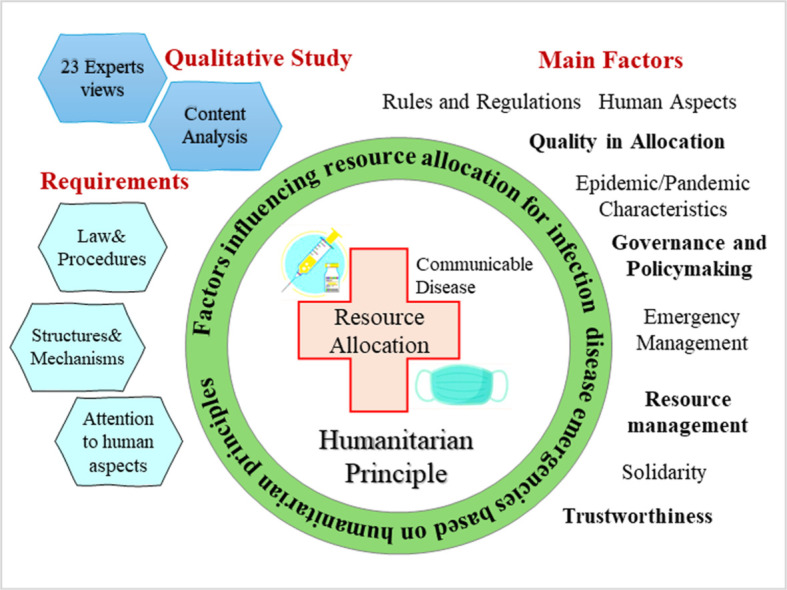

## INTRODUCTION

Infectious diseases have the potential to cause global emergencies.^1^ This situation can create several challenges for public health and emergency management.^2^ One of the most critical aspects of a pandemic response is to ensure access to and provision of resources. Shortages of resources can hinder disease control efforts and exacerbate casualties.^3^ The scarcity of resources can lead to complications in decision-making processes, prioritization, and resource allocation. This can result in dissatisfaction, discrimination, and the emergence of ethical and humanitarian challenges.^4^ For instance, during the COVID-19 pandemic, many people complained about inequalities in resource distribution, where those with better financial conditions had better access to resources.^5^ Hence, resource allocation can pose significant ethical and humanitarian challenges.^6^

Decisions regarding resource allocation and prioritization in such situations are often ambiguous, with less attention given to reducing human suffering and respecting human dignity.^7^ Epidemics and complex emergencies such as wars, conflicts, and political sanctions can further complicate resource management and allocation.^8^

To ensure equitable access to resources for affected communities, various international guidelines and protocols have been developed, such as the SPHERE project and the WHO Health Cluster.^9^^,^^10^ Most of them are based on humanitarian principles. The humanitarian principles encompassing humanity, neutrality, impartiality, and independence are essential to guiding humanitarian action. These principles are backed by two UN General Assembly resolutions (46/182 and 58/114) and provide a framework for all entities engaged in humanitarian work. They articulate humanitarian aid as the provision of life-saving assistance to those in need without making any adverse distinctions.^10^^,^^11^

Despite the established humanitarian standards and guidelines, decision-making processes during epidemics and pandemics are highly complex, particularly when it comes to resource allocation.^12^ As a result, healthcare workers are often burdened with stress and moral dilemmas because of the uncertainties involved in resource allocation decision-making.^13^

Furthermore, studies indicate that the existing guidelines on how to make decisions in the complex conditions of infectious disease emergencies lack specific procedures aligned with humanitarian principles. In these circumstances, using tools and methods for resource allocation can be an effective solution. However, previous studies have paid less attention to the humanitarian principles in resource allocation. Therefore, there is a need to develop resource allocation patterns in line with these principles. To address these challenges, this study was conducted to identify the key factors that influence the principles-based allocation of health care resources during infectious disease emergencies, drawing on the experiences and viewpoints of experts, individuals, and interest groups.

## METHODS

This qualitative study employed content analysis and an inductive approach through in-depth semi-structured interviews. Qualitative research focuses on exploring complex phenomena and understanding the meanings individuals and groups assign to them, emphasizing subjective experiences. It is commonly used in social sciences, psychology, and anthropology to capture the richness of human beliefs, attitudes, and behaviors, providing insights beyond quantitative methods. Data collection includes interviews, observations, focus groups, and document analysis to gather detailed participant perspectives. Given the limited earlier research on humanitarian principles in resource allocation for infectious diseases, this study used semi-structured interviews and content analysis from October to December 2023.^14^^,^^15^

This research was conducted in Iran. The participants in this study are senior and middle managers of the health system, as well as operational managers and health care providers, who are among the personnel working in hospitals and health care centers affiliated with the Ministry of Health, the Red Crescent Society, and some NGOs.

Participants were selected through purposive sampling to ensure diversity.^16^ The sampling from diverse groups enhances our understanding of the phenomenon under study. Participants had varying levels of expertise and represented multiple departments, including hospitals, healthcare services centers, the Ministry of Health, the Red Crescent Society, NGOs, and medical sciences universities, providing a comprehensive perspective that captures both specialized insights and practical viewpoints. The inclusion criteria for the study were having relevant knowledge and experience in resource allocation during epidemics and pandemics, and the exclusion criterion was unwillingness to participate in the research.

The data collection method involved conducting in-depth, semi-structured interviews with the participants. The initial four interviews were unstructured, and after preparing the guidelines, the subsequent 19 interviews were conducted as semi-structured. Each interview lasted between 30 and 120 (average of 53) minutes.

The interviews were conducted in a calm environment and began with simple and general topics and gradually progressed to specific questions based on the participants’ responses. The main content of the questions focused on humanitarian principles and their application in resource allocation during infectious disease emergencies. Some of the questions included, *How do you believe humanitarian principles impact resource allocation in pandemic situations? What factors affect the allocation of resources in accordance with humanitarian principles in infectious disease emergencies?* All the interviews were recorded, and notes were taken; also, the status of the interviewees (memo) were captured to be considered when transcribing the interviews. In addition, the details of the interviewees, the location and the date and time of the interview, their organizational position, and other necessary information were recorded**.**

Because sampling was purposefully done, the number of participants was determined through data analysis to achieve saturation, ensuring that new insights were unlikely to arise. This approach upholds rigor while minimizing unnecessary redundancy. In this study, interviews continued until researchers confirmed that no new concepts emerged, based on the analysis of the last two interviews.

Data analysis was conducted using the five-step Graneheim and Lundman content analysis approach.^14^ All interviews were recorded and analyzed concurrently. Before moving on to the next interview, a preliminary analysis of the previous interview was conducted, and these results were used to guide subsequent interviews.

In this regard, an inductive content analysis method was employed. After transcribing each interview, the text was read multiple times to gain an overall understanding of it. Subsequently, specific sections of the text that contained the key concepts of the codes were then identified. After determining the initial codes, subcategories and categories were extracted based on the similarities and differences of the codes. Finally, themes related to the factors influencing resource allocation according to humanitarian principles were derived. MF conducted the data analysis under the supervision of KJ and MS. To finalize the concept, the research team members discussed the subcategories, categories, and themes.

To uphold trustworthiness considerations such as credibility, transferability, dependability, confirmability, and reflexivity was implemented.^17^ In this regard, factors such as appropriate selection of samples, simultaneous data collection and analysis, establishing good communication with participants, employing triangulation in data collection, participant review, review of observers, and incorporation of supplementary input from the research team were considered.

This study was part of a doctoral dissertation approved by Shahid Beheshti University of Medical Sciences in Tehran, Iran, under the code of IR.SBMU.PHNS.REC.1401.155. To ensure ethical considerations were met, all participants were required to give either oral and written informed consent.

## RESULTS

In this study, 23 participants were interviewed, and their demographic information is presented in Table 1. A total of 1,045 primary codes were initially identified, which were then purified and duplicates were removed. This led to a final count of 373 codes. After data analysis, the factors that affected resource allocation were classified into 9 main themes, 25 categories, and 50 subcategories, as shown in Table 2.

**Table 1:** Participants’ Demographic Characteristics

	Mean/n	Range/percentage
Gender		
Male	14	60
Female	9	40
Age	51.1±8.2	37-65
Work Experience (Years)	22.8±7.6	10-35
Education		
Master's degree	5	21.7
PhD	18	78.3
Occupation		
Nurse	4	17.3
Physician	7	30.4
Humanitarian aid worker	5	22
Disaster manager	4	17.3
Health educator	3	13

The study has identified 9 main themes which are as follows: (1) Rules and Regulations; (2) Quality in Allocation; (3) Human Aspects; (4) Epidemic/Pandemic Characteristics; (5) Governance and Policymaking; (6) Emergency Management; (7) Resource Management; (8) Solidarity; (9) Trustworthiness.

**Table 2:** Results Related to the Factors Affecting the Allocation of Resource by Humanitarian Principles in the Emergencies of Infectious Diseases

Theme	Category	Subcategory	Example of codes
Rules and Regulations	Indicators	Background indicators	Community capacity, insurance coverage rate
		Outcome indicators	Incidence and mortality rate, recovery rate
	Criteria	Prioritization Criteria	Priority by Need Intensity, Priority by Vulnerable Groups
		Decision-making Criteria	Principle of Impartial Selection, Principle of Proportionality
	Standard	Standard	Common Language and Interpretation, Universal Principles
	Protocol and Process	Screening and Triage	Resource Triage Guidelines, Request Screening System
		Health care Service Delivery Processes	Surveillance system, accreditation System
	Laws and Agreements	International	Health Cluster System, United Nations Charter, and UN Treaties
		National	Constitution and Current Government Laws, Anti-Corruption Laws
		Individual and Organizational	Individual and Organizational Professional Behavior Principles, organizational mandate
Quality in Allocation	Integration	Accessibility	Ease of access to resources, equitable access for different groups
		Order and Coherence	Meeting needs in order of priority, organized allocation
	Comprehensiveness	Diversity	Designing and packaging items based on variable needs
		Effectiveness	Comprehensive and accurate vaccination, active disease detection
Human Aspects	Respect	Preserving Human Dignity	Respecting individuals’ self-esteem, instilling the importance of the service recipient
		Respect for Culture and Beliefs	Respect for community traditions and beliefs, local connection and understanding
	Humanity	Humanitarian attitude	Having a uniform view of humans, avoiding causing distress
	Attention to Specific Groups	Attention to vulnerable groups	Attention to deprived groups of society, attention to specific patients
	Non-discrimination	Non-discrimination based on Individual Differences	Avoiding discrimination based on race, ethnicity, language, and gender
		Non-discrimination based on acquired and environmental differences	Disregarding financial status, social status, and nationality
	Ethics	Observing ethical principles	Beneficence and non-maleficence, observing justice
Epidemic/Pandemic Characteristics	Disease Characteristics	Nature of Disease	Emergence and Re-emergence, Seriousness, and Risk Level of the Disease
		Origin of Disease	Intentional and Unintentional, Multiple Origins
		Mode of Transmission	Airborne and Respiratory, Water and Food (Ingestion)
	Characteristics of the Patients	Individual Characteristics	Concurrent and Background Diseases, Age and Age Group
		Contextual Characteristics	Patient's Occupation, Nutritional Status, Financial Status
Governance and Policymaking	Official's Understanding and Knowledge	Awareness	Officials’ Expertise and Knowledge, Understanding of Societal Context
		Commitment	Acceptance of Social Responsibilities, neutrality of health infrastructures
		Support	Organizing the governance system towards pandemic management, attention to areas with less access
	Policymaking	Decision-making	Use of experts’ opinions in Decision-making, making decisions regardless of political inclinations
Emergency Management	Prevention and Preparedness	Prevention of Incidents	Focus on Health and Prevention, Risk Assessment, and Identification of High-Risk Areas
		Education and Culture-building	Providing education to stakeholders, creating a humanitarian culture among donors
		Planning	Planning for Procurement and distribution of essential items, surge capacity program
	Response	Operational Plan	Designing process for identifying affected individuals, activation of hospital surge capacity
		Organizing	Organizing donors and volunteers, organizing and managing gifts and aids
	Recovery and Rehabilitation	Rehabilitation of Individuals	Taking care of the affairs of the families of patients, survivors, and their surroundings, psychological recovery of individuals and patients
Resource management	Human Resources	Personal and Occupational Characteristics	Staff Loyalty and Commitment, Professional Behavior
		Retention	Attention to the psychological and livelihood issues of the staff
	Physical and Information Resources	Infrastructure	Development of infrastructure in pandemic conditions
		Systems and Data	Platforms for resource registration and management, geographic information systems
		Items and Equipment	Vaccines and drugs, beds, and specialized equipment related to the disease
Solidarity	Stakeholder Participation	Participation	Participation in Resource Sharing, Social Engagement
	Coordination and Collaboration	International Coordination	Need for Information from Recipient Countries, Collaboration and Coordination Among All Stakeholders
		International Interactions and Relations	Respect for Countries' Independence, WHO's Authority in International Policymaking
		Coordination Mechanisms	International Coordination Mechanisms, Existence of Agreements with Governments, Companies, and International Organizations
Trustworthiness	Trust in Humanitarian Actions	Transparency	Respecting the independence of all stakeholders, transparency in structures, relationships, processes, guidelines, and actions
		Accountability	Early Detection and Accountability, No Delay in Program and Action Reporting
		Information Dissemination	Joint awareness of service providers and recipients, speed and transparency in information dissemination
	Supervision	Monitoring and Control	Due diligence evaluation before providing aid, control of deviation from humanitarian goals
		Supervisory Mechanisms	Supervisory mechanisms of supervisory organizations (WHO, Red Cross, etc.), the existence of deterrent mechanisms for compliance with principles

The factors listed in Table 2 can aid in the implementation of humanitarian principles. The study suggests that the application of humanitarian principles during infectious disease emergencies can be discussed at the healthcare service level, including hospitals and health centers; the community and public health levels are governed by countries; and the international level that stakeholders such as countries, donors, international organizations, and health supply companies influence the implementation of these principles.

Taking humanitarian principles into account requires the establishment and implementation of regulations and rules. These may include indicators such as service coverage rates, incidence and mortality rates, and the number of preserved life years. In addition, prioritization criteria and decision-making principles such as the principle of proportionality and priority based on the intensity of need are among the relevant considerations. Also, protocols and processes such as triage guidelines and health service delivery processes can impact humanitarian principles. Other important factors in this regard include laws and regulations. International conventions and treaties, human rights, countries' constitutions, and anti-corruption laws all influence the attainment of humanitarian principles. *” Within the country, a COVID scientific committee was formed and began to develop protocols. The protocols were influenced by conflicting interests, personal approaches, and political orientations; also, there were challenges in accepting the protocols and guidelines.” P4*

Integration and the comprehensiveness of actions can greatly facilitate the attainment of humanitarian principles. Easy access to resources and meeting the minimum needs in emergencies facilitate access to resources. Furthermore, proportionality in allocation and orderly allocation show consistency in allocation. Achieving comprehensiveness requires diversity and having choices in items such as vaccines and medicines. In terms of effectiveness in allocation, actions such as accurate vaccination, active disease detection, and provision of essential services for free, are needed. *“Suspending certain rules in emergencies, for example, if someone comes can receive free essential services. In the early stages of the COVID pandemic, this happened to some extent, making COVID diagnosis free, which helped a lot.” P5*

Observing respect and preserving the dignity of individuals, as well as respecting their culture and beliefs, are essential influential factors in humanitarian allocation. Humanitarianism is manifested through actions such as prioritizing individuals' lives, providing services to alleviate suffering, non-discrimination, and addressing psychological and human aspects of quarantine. Paying attention to vulnerable groups and individuals with greater needs, such as children, pregnant women, the elderly, and those with underlying diseases, and adhering to ethical principles such as respecting personal autonomy, justice, and beneficence, non-maleficence are other related human aspects of allocation. *“In the domain of allocation in infectious diseases, the art of balancing benefits and harms is crucial, entering it all at once aside from ethical tensions, there is nothing else, conflicts of interest must be managed.” P12*

During an epidemic and pandemic, the characteristics of the disease and the attributes of those affected significantly impact management actions and resource allocation. The emergence and re-emergence of a disease greatly affects preparedness and coping knowledge. The severity and level of disease risk, as well as the target organ of the disease, influence the type of required resources and their provision. *“A disease that occurs requires significant control by systems, whether transmitted through respiratory routes, ingestion, and based on that, humanitarian services are provided to control the disease cycle.” P9*

Officials' comprehension, knowledge, and decision-making affect policymaking in resource allocation for infectious diseases. Humanitarian allocation requires officials’ support in organizing governance structures, paying attention to less accessible areas, supporting migrants and refugees, vaccine and drug production, striking a balance between domestic and foreign needs, and using effective officials. *“Perhaps if you ask many officials and managers, many of them do not know these principles. Then the lack of awareness and commitment of officials leads to humanitarian issues being influenced by political, security, and other tendencies.” P20*

Emergency management involves a comprehensive approach that covers prevention, preparedness, response, and recovery from pandemics and epidemics. It entails educating all stakeholders, including officials, humanitarian organizations, and the public, on humanitarian principles while fostering a culture of social awareness. Planning for essential items, the supply chain, and procurement is also critical. *"The lack of awareness of officials, whether from universities, hospitals, clinics, and NGOs leads to the failure to adhere to principles. Therefore, everyone should receive proper education and cultural awareness." P23*

Human resources and resources such as infrastructure systems and data facilitate the implementation of humanitarian principles. The psychological and livelihood issues of staff, the work and rest cycles, and the prioritization of health care staff in resource allocation are important. Attention to health care infrastructure, platforms for recording and managing resources, and geographical information systems (GIS), as well as the availability of items such as vaccines, drugs, and diagnostic kits, can facilitate humanitarian actions in pandemics. *“In the early days of COVID-19, we had a shortage of N95 masks for personnel, and there were also problems with their use. We had a shortage of human resources and a shortage of beds. Laboratory personnel were forced to work long hours.” P16*

Infectious diseases can spread beyond specific geographical and borderlines. Therefore, solidarity, participation, coordination, and collaboration among various stakeholders facilitate humanitarian actions. International coordination mechanisms are essential for enhancing solidarity. Requesting assistance requires using international processes and engaging with organizations such as the United Nations, the World Health Organization, and the Red Cross. *“To be able to use international and national services, it is necessary to strengthen communications with the United Nations, the World Health Organization, and other organizations, and even make suggestions for international Legislation to receive health services. Our country needs to work on this issue.” P18*

Trust in the humanitarian allocation of resources requires transparency, accountability, information dissemination, supervision, and control. Transparency in structures, relationships, processes, guidelines, and actions, financial and social impartiality, respect for stakeholders' independence, and avoiding delays in providing accurate descriptive-analytical and financial reports will strengthen trust. *“The donor and the humanitarian organization must be able to conduct a thorough review of the organization and institution receiving aid to see if it has transparency, does not have administrative problems, and does not have problems related to receiving aid.” P8*

## DISCUSSION

This study identified the factors influencing the allocation of health resources during infectious disease emergencies, grounded in humanitarian principles as perceived by experts. To evaluate the effectiveness of these principles, key indicators such as health metrics, mortality rates, and morbidity rates serve as benchmarks. It is also crucial to consider international, national, and organizational laws and agreements, as they play a vital role in this area. Van Hout and Wells, in their study, identified the Universal Declaration of Human Rights, the right to health, and the fundamental principles of international humanitarian organizations and national laws as the basis for humanitarian actions, which was also discovered in this study.^18^

The study's findings on prioritization criteria are consistent with previous research, indicating that personnel involved in operations should be prioritized for resource allocation.^19^ This study has identified specific criteria for resource allocation, highlighting the importance of prioritizing based on the intensity of need, as well as the principles of efficiency, non-maleficence, and equality. These criteria are crucial for ensuring that resource allocation aligns with humanitarian principles.^20^^,^^21^

Preserving individuals’ dignity and addressing vulnerable groups is crucial to the human aspects of allocation. Attention should also be given to the cultural context, local beliefs, and societal requirements in resource allocation decisions, as highlighted in Walker's study.^22^ Discrimination is a deterrent to the humanitarian nature of resource allocation. In pandemics, non-discrimination does not necessarily mean equality in its common sense. When the allocation criterion is solely humanitarian, addressing pain and technical issues while considering ethical concerns, the outcome of resource allocation is accompanied by non-discrimination, meaning equality for equal needs. Discrimination can pose a significant challenge to disease control measures, as shown in similar research.^23^^,^^24^

In addition, attention to ethics, along with attention to humanitarian principles, is necessary. Ethics is part of the human aspects of resource allocation in epidemics. Various articles have sought to address the issue of ethics,^25^ and the experiences during the COVID-19 pandemic have further highlighted the importance of ethical considerations in resource allocation.^26^ Infectious diseases manifest in different types, each having unique effects on individuals and involving different organs. Some studies show that certain diseases, like COVID-19, had a lesser impact on children but significantly affected individuals with underlying diseases. Also, because of the lack of knowledge, information, vaccines, and medicines in the early stages of the pandemic, various efforts to control the disease were ineffective.^27^

Governments are a major component of policymaking and implementing humanitarian principles, and their knowledge of these principles and their commitment have a great impact on how resources are allocated. The results of a study conducted in Malaysia also emphasized the necessity of government support in controlling the disease, paying attention to quarantine problems, and the like.^28^

Implementing the emergency management cycle approach has a beneficial impact on resource allocation.^29^^,^^30^ Similar studies have underscored the significance of preparation and planning in minimizing the repercussions of resource allocation. Currently, readiness for any interventions, exemplified by the concept of disease X, has garnered attention from the World Health Organization and other entities. This highlights the international community's preparedness to address future occurrences.^31^

In the emergency management cycle approach, readiness for response and organization of coping strategies before the onset of a pandemic are planned. During the pandemic, contingency plans are formulated, and existing plans are updated. The mentioned experiences concerning the implementation of the emergency management cycle approach in Iran encompass actions such as establishing technical committees to derive decision-making criteria for addressing COVID-19, restructuring command structures, activating hospital surge capacity, and organizing volunteers in the response phase. In addition, there was a noticeable need for the recovery and rehabilitation of people and patients from the effects of COVID-19.^32^

Resources management to achieve humanitarian principles is crucial, with the preservation of human resources being a key component of actions in this regard. Participants in this study highlighted various challenges faced by the health care workforce in combating COVID-19, such as stress from their own and family members’ exposure to COVID-19, difficulties in using personal protective equipment for extended periods, high workload, problems such as pregnancy and working concurrently in an infected environment, and repeated exposure to COVID-19. Therefore, implementing humanitarian principles without considering the health care personnel and their challenges would be incomplete. This issue has also been noted in many studies.^33^^,^^34^

In addition to focusing on human resources, the availability of essential items for coping with diseases affects the application of humanitarian principles. Problems with hospitalization because of the high number of COVID-19 cases and limited hospital bed capacity were observed in this regard. Furthermore, issues such as the black market for items like personal protective equipment and medications, sales at inflated prices, profiteering, and the sale of counterfeit vaccines were among the problems resulting from resource shortages and improper management.

Another key aspect of resource allocation is transparency in actions, responsiveness, information dissemination, and oversight. Even if these principles are observed, a lack of transparency and responsiveness can pose challenges to individuals’ perceptions of the humanitarian nature of resource allocation. This issue has been emphasized in many articles.^22^^,^^25^^,^^26^^,^^35^ Trust and transparency lead to impartiality, neutrality, and independence to a significant extent. This means that without a transparent system, understanding the adherence to these principles is severely affected. Therefore, in societies and systems lacking transparency, one cannot speak of the humanitarian nature of actions. In the context of solidarity, McGowan at al. believe that community participation, stakeholder collaboration and coordination, and community-centered oversight can facilitate the humanitarian nature of actions.^36^ Solidarity is an emerging principle in the humanitarian context, which has been addressed in similar research and was also extracted in this study.^37^ The solidarity among various stakeholders in the context of infectious diseases, established through national and international cooperation and coordination, significantly influences disease control and a global pandemic response.^38^ This was evident in the COVID-19 context with the COVAX system designed to facilitate coordinated mechanisms.^39^

## CONCLUSION

The importance of applying humanitarian principles in the allocation of resources during infectious disease emergencies is emphasized in the latest study. To ensure efficient implementation, attention must be given to both functional, structural, and procedural issues, such as protocols, processes, laws, and organizational and coordination structures, as well as content-related issues, such as respecting human aspects and ethical principles. It is crucial to consider these principles at various levels, both within and outside countries.

The study highlights that a lack of awareness and commitment among individuals involved in resource allocation is one of the barriers to implementing humanitarian principles. Therefore, education and cultural promotion before pandemics are effective in this regard. In addition, there is a need for oversight and control to combat phenomena such as black markets and hoarding in the resource access section. For future research, it is suggested to explore the challenges of implementing humanitarian principles in infectious disease pandemics, with a focus on COVID-19.
